# Parliament and the Professions

**Published:** 1921-07-16

**Authors:** 


### Parliament and the Professions.

### Pensions Ministry Medical Officers

Mr. A. T. Davies asked a question of the Minister of Pensions as to the percentage of doctors employed by the Ministry who have had overseas experience. Major Tryon, who answered, said that he was unable to state the exact number, but it was considerably more than 60 per cent. Preference was given wherever possible to men who have served overseas, but other important qualifications, such as experience in special classes of disease, must often be taken into consideration.

### Scarlet Fever.

Sir Alfred Mond stated that in the Metropolitan area there are approximately 2,000 cases more of scarlet fever in the hospitals of the Metropolitan Asylums Board than there were in the same month last year. Such epidemics tend to recur at intervals of seven years; the causes of this periodicity are being closely studied, but have not yet been fully elucidated. In answer to another question, as to the effect of the drought upon scarlet fever, diphtheria, and similar diseases in the valley of the Lea, it was stated that the increase during the last few weeks in that district is not disproportionate to the increase in other parts of the Metropolitan area. There is no clear evidence that epidemic spread of these diseases in the neighbourhood of the river can be caused by river pollution. The Minister is taking appropriate steps to ensure that sewage discharged into the river Lea is properly treated and purified before such discharge.

### Tuberculosis.

The Minister of Health stated that an official report is being constructed giving particulars of the number of cases notified in England and Wales during the years 1914 to 1920. These figures show a considerable reduction in the number of notified cases during the last two years, the decrease in mortality being 58,073 in 1918 to 46,312 in 1919, and 42,545 in 1920. Sir Alfred Mond submitted a table showing that cases of pulmonary tuberculosis had declined from 77,109 in 1914 to 57,844 in 1920, while other forms of tuberculosis had decreased from 23,888 in 1914 to 15,488 in 1920.

### Psycho-Analysis.

In answer to a question, the Minister of Pensions stated that satisfactory results have been obtained by treatment by psycho-analysis, which, however, is in no sense compulsory, as an essential element of the treatment is the patient's willing co-operation. Treatment is always given by registered medical practitioners who are specially qualified by previous training or experience. Members of the House of Commons can be afforded an opportunity of seeing the hospital methods of this matter.

### Dublin Hospitals

Mr. T. W. Brown, answering a question put by Mr. Devlin to the Chief Secretary for Ireland, stated that he was aware of the figures in the report of the Board of Superintendents of the Dublin hospitals for the year ending March 1, 1920. With regard to the financial position of the hospitals of that city the deficit was shown as follows: Stevens' Hospital, £7,946; Meath Hospital and County Dublin Infirmary, £6,688; Cork Street Fever Hospital, £5,843; Rotunda, £5,014; Coombe, £4,355; Royal Victoria Eye and Ear, £7,500; Royal Hospital for Incurables, £7,279. He was unable to make any further statement with regard to any method of relieving the financial position of these hospitals.

